# The Microstructure Formation of a Protective Oxide-Scale Layer on Small-Diameter FeCrAl Fibers

**DOI:** 10.3390/ma15217444

**Published:** 2022-10-24

**Authors:** Abdullah A. Alazemi, Osama M. Ibrahim

**Affiliations:** Mechanical Engineering Department, Faculty of Engineering and Petroleum, Kuwait University, P.O. Box 5969, Safat 13060, Kuwait

**Keywords:** FeCrAl fibers, small fiber size, thermal oxidation, multistage heat treatment, oxide-scale layer

## Abstract

FeCrAl fibers, at high temperatures, form a protective oxide-scale layer dominated by aluminum oxide on the surface to prevent further oxidation of the base metal alloy. This study investigates the effects of heat treatment on the microstructure formation of the oxide-scale layer on small-diameter FeCrAl fibers, 12 and 17 µm, produced using a bundle drawing process. The morphology examination and chemical analyses of the small-diameter fibers exhibit the microstructure and chemical compositions of the surface and cross-section areas, revealing a distinctive interface layer with a high aluminum concentration between the base metal and the oxide-scale layer. Furthermore, thermogravimetric analysis results show that the 12 µm fibers have about a 60% higher oxidation rate than the 17 µm fibers—caused by the high outward diffusion of aluminum to the surface of the fibers due to their high surface-area-to-weight ratio. Consequently, the high growth rate of the nonuniform oxide-scale layer and the limited aluminum reservoir of the 12 and 17 µm diameter fibers lead to faster depletion of aluminum from the base metal alloy—limiting the lifetime and durability of the smaller-diameter fibers in high-temperature applications.

## 1. Introduction

Metal fibers have gained enormous attention in the research community and industry [1]. Compared with glass and carbon fibers, which offer good mechanical properties and low weight, metal fibers have high thermal corrosion resistance at high temperatures and high electrical and thermal conductivity [2]. The focus of this paper is on sintered metal fibers that are made of iron–chromium–aluminum (FeCrAl) alloy. FeCrAl fibers are widely known for their high oxidation resistance at elevated temperatures. Therefore, they are used in specific applications, such as heating elements, surface burners, high-temperature filtration systems, and high surface area substrates as catalyst support. However, one factor limiting the effectiveness and lifetime of FeCrAl fibers is the depletion of the aluminum content from the base metal FeCrAl alloy resulting from the high aluminum diffusion rate, leading to the discontinuous growth of the surface protective oxide layer. Therefore, it is crucial to study the microstructure formation of the oxide-scale layer and the rate of aluminum diffusion of small-diameter FeCrAl fibers at elevated temperatures to predict their physical and chemical stability under multistage heat treatment to advance their performance in high-temperature applications.

Metal fibers can be produced using different methods, such as bundle drawing, foil shaving, machining, and melt spinning. The bundle drawing process produces long metal fibers with high ultimate tensile strength, relatively uniform diameter, and smooth surface. In this process, several wires are bundled together and covered with a tube. Afterward, the bundled wires are drawn simultaneously through multiple dies to reduce their diameters. The process is repeated until the desired fiber diameter is reached. The covering tube is then chemically removed, and the long continuous metal fibers are acquired. The bundle drawing process can produce metal fibers several kilometers in length. However, this process is only economically feasible to manufacture metal fibers with diameters less than 50 µm [1,2].

Many studies have examined the formation of the protective oxide-scale layer on the surface of FeCrAl rods, strips, foils, or cuts of standard specimens [3,4,5,6,7,8,9,10,11,12,13,14,15]. Quadakkers et al. [8] demonstrated that parabolic scaling kinetics for thin components of FeCrAl alloys leads to underestimating their actual lifetime limit. They showed that α-alumina growth at 900–1000 °C follows subparabolic rather than parabolic kinetics. The formation of metastable/stable phases and the growth rate of the oxide-scale layer were studied at various isotherms and exposure times in single-stage heat treatment. A multistage heat treatment (MSHT) process was also considered, where different temperatures for a specified exposure time are designed to accelerate the scale layer’s growth and develop interesting morphology dominated by whiskers and a platelet-like surface microstructure [16,17]. Abnormally high growth rate, void formation, and oxide-scale layer breakdown were also studied [18,19,20,21]. Whittle and Stringer [22] presented various theoretical models explaining the effect and importance of rare earth elements in reducing the growth rate and improving the adhesion between the scale-oxide layer and the base metal alloy. Various experimental studies have investigated the effects of rare earth elements on oxidation behavior [23,24,25,26,27,28]. However, the literature survey reveals few technical papers on FeCrAl fibers [17,29,30,31,32,33,34,35,36]. Li et al. [31] and Fornasiero et al. [32] investigated FeCrAl fibers as a substrate and support of catalysts. Fei et al. [33] examined how metastable alumina platelets and whiskers grew on FeCrAl fibers at temperatures between 800 and 900 °C. They concluded that the accelerated growth rate of Al_2_O_3_ can deplete the aluminum content in the base metal alloy. The MSHT thermal oxidation process was performed by Samad et al. [17] on FeCrAl sintered metal fibers as catalyst support. Their study focused on forming α-Al_2_O_3_ for palladium catalyst support during an MSHT thermal oxidation process. Their results showed that α-alumina was the primary phase for the oxide scale on the fiber’s surface achieved by one cycle of MSHT. It is recognized that the MSHT process decreases the growth period of the oxide scale on FeCrAl fibers; at the same time, it reduces the aluminum reservoir and limits the lifetime of the base metal FeCrAl alloy [33].

Ibrahim et al. [36] conducted a study to examine the effects of MSHT on FeCrAl sintered metal fibers 40 µm in diameter. In their research, the oxide-scale layer was developed using an MSHT cycle. Three samples were evaluated: A baseline sample, Sample 1, was kept without thermal oxidation as received. Sample 2 was subjected to one MSHT cycle, while Sample 3 was subjected to multiple MSHT cycles. The results of Ibrahim et al. [36] for one MSHT cycle were similar and confirmed the results presented by Samad et al. [17]. However, after multiple MSHT thermal oxidation cycles, the oxide-scale layer evolved into a different microstructure and revealed diffraction pattern peaks of a stable α-Al_2_O_3_ and crystalline Cr.

The investigation of Ibrahim et al. [36] was focused on large-diameter FeCrAl fibers. Small-diameter FeCrAl fibers offer a high surface-area-to-volume ratio, making them more suitable for special applications, such as a catalyst substrate and in hot gas filtration [37]. However, the high surface-area-to-volume ratio also makes them susceptible to high aluminum diffusion, which could lead to the depletion of the aluminum reservoir from the base metal alloy—affecting the growth rate and the microstructure of the protective oxide layer. This paper aims to study the microstructure formation of an oxide-scale layer during single and multiple MSHT cycles on small-diameter FeCrAl fibers. Two fiber diameters were considered in this investigation: 12 and 17 µm. All FeCrAl fibers examined in this study were produced using a bundle drawing process.

## 2. Experimental Methods

### 2.1. Sintered Metal Fibers: Material and Sample Preparation

In this study, FeCrAl sintered fibers with two different diameters (12 and 17 μm) were used to investigate the effect of fiber size on the thermal oxidation process. Before testing, all samples were cleaned using alcohol sonication for about 30 min and then dried in an oven at 120 °C for 1 h. The elemental chemical compositions of the FeCrAl sintered fibers used in this study are summarized in Table 1.

In the current investigation, the samples were examined at different MSHT cycles to carefully study the formation of the oxide layer on the surface of the FeCrAl fibers. As received, the two sintered fibers were analyzed first without heat treatment. Then, they were analyzed after one MSHT cycle and, finally, after multiple MSHT cycles. Each MSHT cycle is a 4 h heat treatment process in dry air at step-up temperatures: at 930 °C for 1 h, then 960 °C for 1 h, and finally, at 990 °C for 2 h, as described in Table 2. In multiple MSHT cycles, dry air was used to cool the sample down to 25 °C for 10 min before restarting the next MSHT cycle.

### 2.2. Characterization Techniques

The morphology of the FeCrAl fibers was inspected using a field-emission scanning electron microscope (SEM), Model JEOL JSM-7001F, acquiring high-resolution images of the fibers’ surface and cross section. Furthermore, chemical analyses of FeCrAl fibers were conducted using energy dispersive X-ray spectroscopy (EDS). To examine the oxide layer’s development on the FeCrAl fibers’ surface, a cross section of the fibers after heat treatment was prepared via a cross-section polisher, JEOL SM-09010, and subsequently, SEM and EDS analyses were performed. The cross-section polishing using ion beam milling was used to prepare cross sections of the samples at cold mounting conditions. Sintered fibers of each sample were placed in the holder and cross-sectioned. Then, a near-perpendicular high-quality cross section of one of the multiple fibers was chosen for further analysis using SEM and EDS.

Thermogravimetric analysis (TGA), using a TA Instruments TGA Q50 analyzer, was performed on the FeCrAl fibers to measure their mass variation, due to the development of a thermal oxide layer on their surface, during single and multiple MSHT cycles. The TGA chamber was purged with dry air for 1 h before the test. Then, about 20 mg of each sample of the fibers was loaded into a ceramic crucible pan with a 70 µL capacity within the TGA instrument. Before conducting any MSHT cycle, the temperature was raised to 930 °C at a 50 °C/min heating rate.

Furthermore, X-ray diffraction (XRD) analyses were performed using an X-ray diffractometer (Rigaku, Japan) operating at an acceleration voltage of 45 kV to identify the crystalline formation on the fibers’ surface of the two samples after different heat treatment conditions.

All tests were repeated several times to ensure the repeatability and reliability of the obtained experimental results. 

## 3. Results and Discussion

### 3.1. Thermogravimetric Analysis

**Figure 1:**
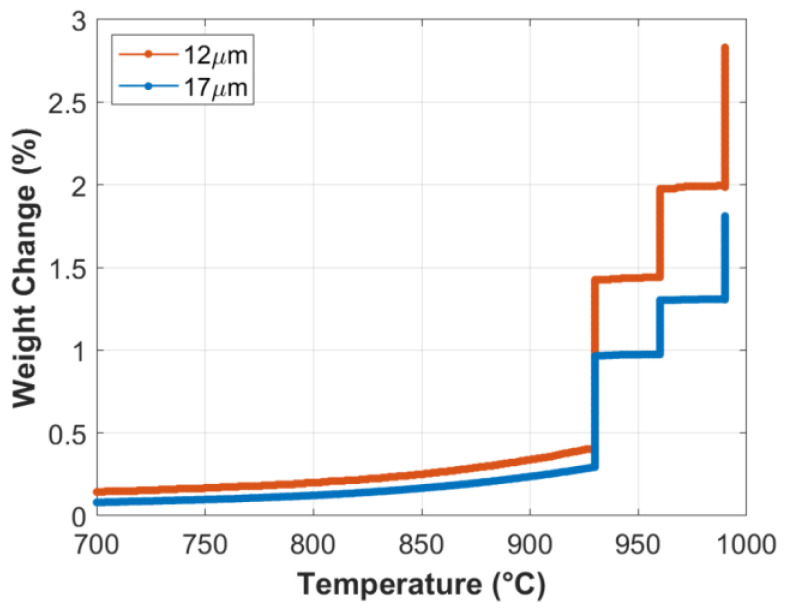
Percentage weight change of the 12 and 17 μm FeCrAl fibers versus temperature during the first MSHT cycle.

TGA analyses were conducted to investigate the formation rate of the oxide layer on the surface of small-diameter FeCrAl fibers. At the start of the TGA analysis, the temperature was elevated to 930 °C from room temperature at a 50 °C/min heating rate, before executing a complete MSHT cycle, as described in Table 2. Percentage weight gains at each stage of the first MSHT cycle and for each fiber size are shown in Figure 1 and summarized in Table 3. During the first stage, weight gains of 1.0% and 0.7% were measured, while during the second stage, the weight gains were 0.6% and 0.3%; and finally, during the third stage, the weight gains were 0.8% and 0.5% for the 12 and 17 μm fibers, respectively. The weight gained by the different FeCrAl fibers during the heat treatment process is attributed to the formation and growth of the thermal oxide layer on the surface. The results show that the weight gain increases as the fiber diameter decreases. It is also worth noting that the first stage of MSHT has the highest rate of weight gain per hour for the two fiber sizes, while the third stage has the lowest.

**Figure 2:**
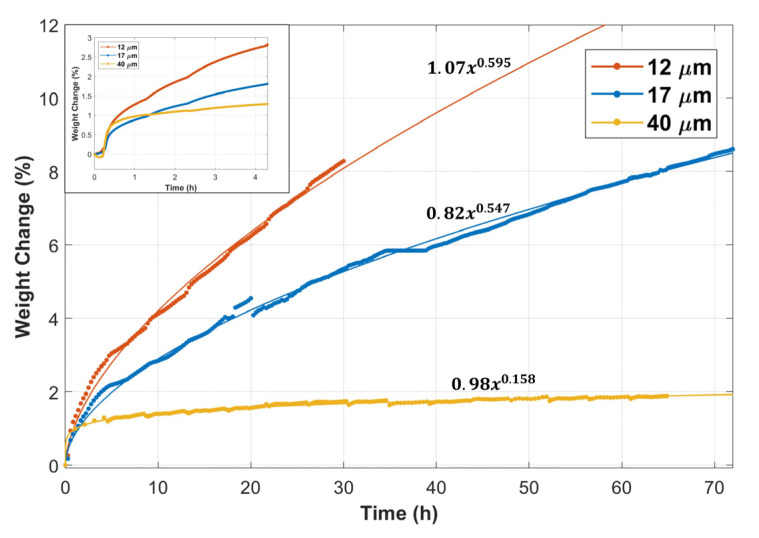
Percentage weight change of the 12, 17, and 40 μm FeCrAl fibers versus time during multiple MSHT cycles. **Inset:** Percentage weight change of the fibers during the first MSHT cycle.

Figure 2 displays the weight change versus time of the 12 and 17 μm fibers, compared with the 40 µm fibers’ TGA results obtained from Ibrahim et al. [36]. The inset in Figure 2 shows the weight change versus time during the first complete MSHT cycle. The percentage of weight gain in the first 20 min was nearly 0.5% for all fibers. However, after 20 min, the rate of weight gain started to vary for the different fibers. The 12 μm fibers had a higher weight gain rate than the 17 and 40 μm fibers. The total percentage of weight gain after one complete MSHT cycle was 2.8%, 1.8%, and 1.3% for the 12, 17, and 40 μm fibers, respectively. Figure 2 also shows the percentage of weight gain as a function of time during multiple MSHT cycles for the different FeCrAl fibers. A power law curve fit was applied to experimental data, indicating that the thermal oxidation process follows a sub-parabolic time dependence. This subparabolic relation during the oxidation of FeCrAl material was observed by several researchers [8,24,28]. The difference in the rate of weight change for all FeCrAl fibers increases with time. Ibrahim et al. [36] demonstrated that, after 18 MSHT cycles, large-diameter fibers (40 μm) almost reached a plateau value of weight gain of about 2%. In contrast, the 12 and 17 μm fibers continued to increase in weight gain with heat treatment time. However, the rate of weight gain for the 12 μm fiber was about 60% higher than the 17 μm fiber.

This behavior can be attributed to variation in the surface area per gram for the different size fibers, as shown in Table 4. It should be noted that the surface areas were obtained by assuming 1 g of FeCrAl in the shape of a long fiber with a circular cross-section area. It is evident from Table 4 that the 12 μm fiber has more than three folds of the surface area per gram of the 40 μm. The high surface area of the smaller-diameter fibers allows for more diffusion of aluminum to the surface of the fibers to form the Al_2_O_3_ oxide layer during the heat treatment cycles, which can explain the high weight gain of the 12 μm fibers compared with the 17 and 40 μm fibers. In addition, the cyclic-oxidation conditions result in a rapid alumina growth rate and phase change of the alumina scale. Therefore, internal stresses are expected to develop on the alumina layer due to volume and thermal expansion variations. Furthermore, the diameter of the fiber and its curvature could affect the stress field and, consequently, the alumina growth rate and the adhesion to the base metal alloy. Quantifying the stress field and its dependence on the curvature, steeper depletion profiles, and other factors are outside the scope of this study and require further investigation.

### 3.2. SEM Micromorphology and EDS Spectroscopy

The micromorphological examination of the FeCrAl fibers was performed via SEM. Furthermore, the fibers’ surfaces and cross-section areas were chemically analyzed using EDS. The current study examined two fiber size samples of the FeCrAl fibers: (1) before heat treatment—as received; (2) after one MSHT cycle; and finally; (3) after multiple MSHT cycles.

#### 3.2.1. Baseline—Before Heat Treatment—As Received

**Figure 3:**
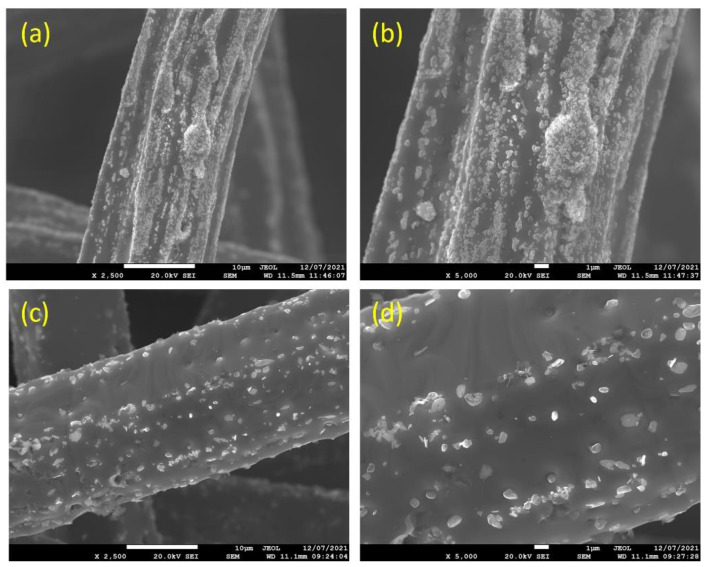
Without heat treatment, SEM micrographs of the FeCrAl fibers at different magnifications: (**a**) ×2500 and (**b**) ×5000 for the 12 μm fibers; (**c**) ×2500 and (**d**) ×5000 for the 17 μm fibers.

Figure 3a–d depicts SEM micrographs of the 12 and 17 μm fibers, without heat treatment, at different magnifications. It can be seen from Figure 3a,b that the 12 μm fibers have a random distribution of irregular-shaped particles (nodules) over the surface of the fibers. Like the 12 μm fibers, the 17 μm fibers, shown in Figure 3c,d, have a random distribution of nodules over the surface; however, at a lower rate compared with the 12 μm fibers. It should be noted that during the manufacturing of metal fibers, the 12 μm fiber is exposed to additional drawing stages compared with the 17 μm fibers, resulting in more and larger nodules and deeper longitudinal grooves on the surface.

**Figure 4:**
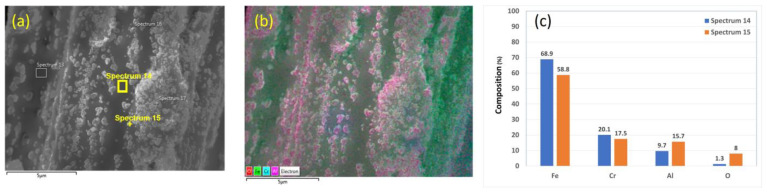
A 12 μm FeCrAl fiber without heat treatment: (**a**) SEM micrograph image, (**b**) EDS layered image, and (**c**) EDS scans of Spectrum 14 and Spectrum 15.

Chemical analyses of the 12 μm fibers without heat treatment are presented in Figure 4a–c. Figure 4b shows an EDS layered image of a 12 μm fiber. The nodules in the SEM images contain high aluminum (Al) and oxygen (O) concentrations. This finding is supported by the EDS scans performed on two different points on the fiber’s surface, as shown in Figure 4c. In addition, the EDS scan completed on a nodule on the fiber’s surface contains significantly higher Al and O concentrations than the EDS scan on an area free from those particles, as shown in Figure 4c.

**Figure 5:**
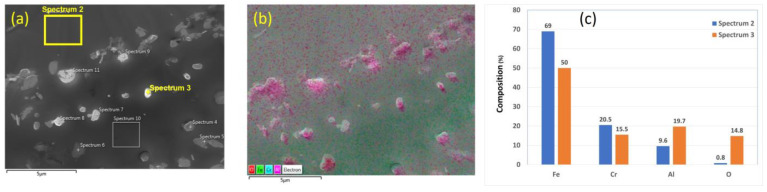
A 17 μm FeCrAl fiber without heat treatment: (**a**) SEM micrograph image, (**b**) EDS layered image, and (**c**) EDS scans of Spectrum 2 and Spectrum 3.

On the other hand, the chemical analyses of the 17 μm fibers without heat treatment are presented in Figure 5a–c. Similar findings on the chemical composition of nodules on the surface of the FeCrAl fibers are obtained. These results confirm that no complete oxide film was formed on the surface, and only Al_2_O_3_ nodules were formed on the surface of the different FeCrAl fibers as received and before applying any heat treatment.

#### 3.2.2. After One MSHT Cycle

**Figure 6:**
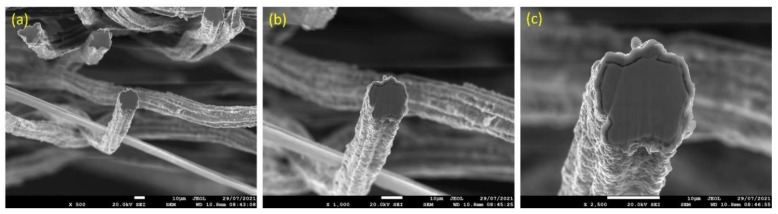
SEM micrographs of cross section of 12 μm FeCrAl fibers, after one MSHT cycle, at different magnifications: (**a**) ×500, (**b**) ×1000, and (**c**) ×2500.

**Figure 7:**
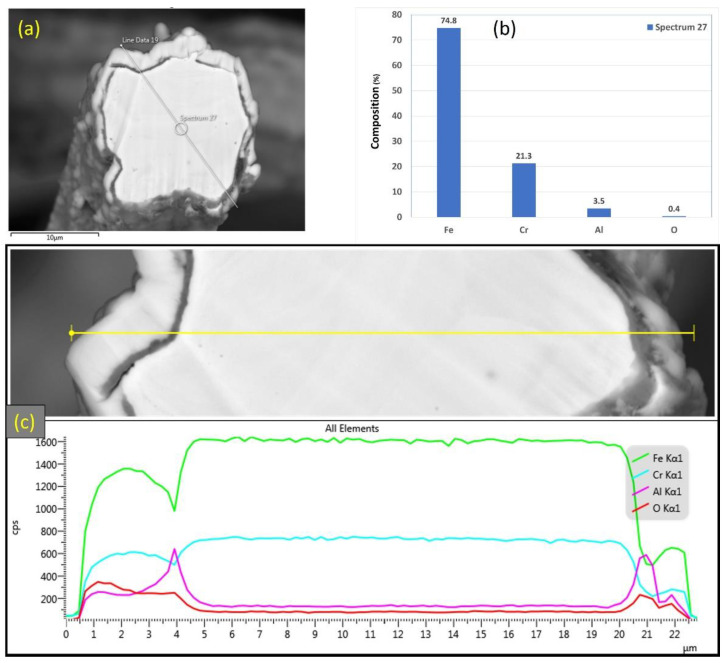
(**a**) SEM micrograph of a cross section of the 12 μm FeCrAl fiber after one MSHT cycle, (**b**) EDS scan at the center of the fiber, (**c**) the characteristic X-ray counts along the specified line scan (Line Data 19) marked in (**a**).

After one MSHT cycle, a cross-section polisher was utilized to obtain a cross section of the different FeCrAl fibers and then carefully examine them using SEM and EDS. Figure 6a–c displays SEM micrographs to varying magnifications of a 12 μm fiber cross-section area after performing one MSHT cycle. It can be seen from Figure 6c that the 12 μm fiber developed a layer (~1.5 μm) over the surface of the fibers. The 12 μm fiber’s cross-sectional area clearly shows the longitudinal groove’s effects on distorting the fiber’s circular shape. EDS analyses were performed on the cross-section area of the 12 μm fiber, as shown in Figure 7a–c. Figure 7b shows the EDS spectrum for a small circular area near the center of the 12 μm fiber. The results revealed a 3.5% aluminum concentration, lower than the base FeCrAl concentration of around 5.76%, as listed in Table 1.

Additionally, EDS analyses were performed on a line covering the entire diameter of the fiber with its oxide layer to qualitatively compare the concentration of the Fe, Cr, Al, and O elements within the fiber, as shown in Figure 7c. The EDS results along this line reveal a high concentration of Al and O along with a low concentration of iron (Fe) at the oxide layer region on both edges of the fiber. Moreover, a drop in the concentration of Al is observed within the inner surface of the fiber. These results show the outward diffusion of aluminum from the base metal alloy to the outer surface to form an oxide layer, resulting in the sample weight gain observed in the TGA analysis results of Figure 2.

**Figure 8:**
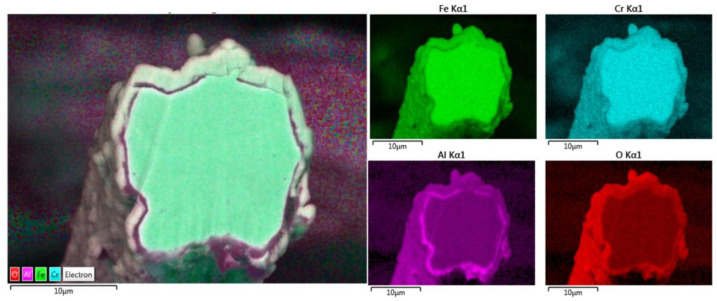
Elemental EDS mapping of Fe, Cr, Al, and O of a 12 μm FeCrAl fiber after one MSHT cycle.

Further chemical analyses were performed of the entire cross-section area of the 12 μm fiber after one MSHT cycle using elemental EDS mapping of Fe, Cr, Al, and O, as shown in Figure 8. The elemental mapping of the base metal after one MSHT cycle reveals a homogenous FeCrAl alloy. Furthermore, the elemental mapping of Al and O also confirms the formation of an oxide layer around the fiber.

**Figure 9:**
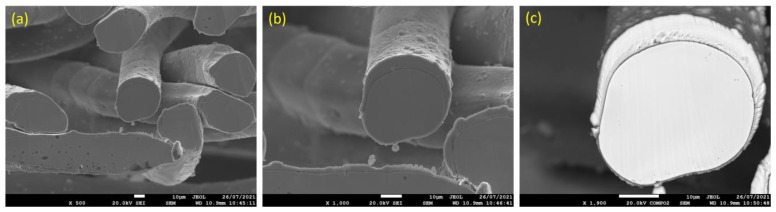
SEM micrographs of a cross section of 17 μm FeCrAl fibers, after one MSHT cycle, at different magnifications: (**a**) ×500, (**b**) ×1000, and (**c**) ×1900.

**Figure 10:**
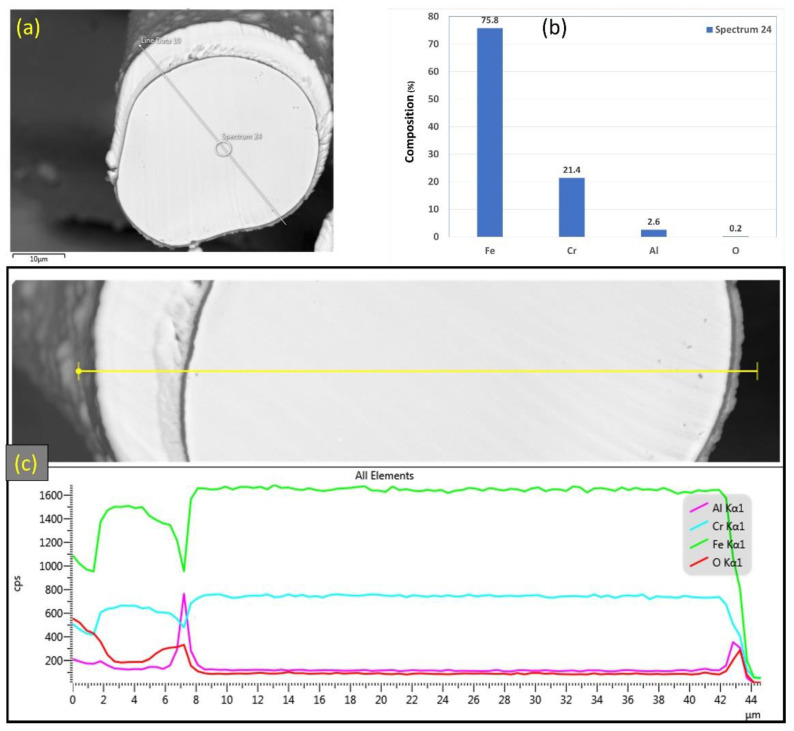
(**a**) SEM micrograph of a cross section of the 17 μm FeCrAl fiber after one MSHT cycle, (**b**) EDS scan at the center of the fiber, (**c**) the characteristic X-ray counts along the specified line scan (Line Data 10) marked in (**a**).

Similarly, Figure 9a–c displays SEM micrographs at different magnifications of a cross section of a 17 μm fiber after one MSHT cycle. The results show that the 17 μm fiber formed an oxide layer with a nonuniform thickness on the surface (average thickness of ~2 μm). Compared with the 12 μm fiber in Figure 6c, the 17 μm fiber in Figure 9c shows a relatively uniform circular cross-sectional area of the base metal fiber after one complete MSHT cycle. EDS analyses were conducted on a 17 μm fiber, as shown in Figure 10a–c. Figure 10b presents the EDS spectrum for a small area near the 17 μm fiber center. The obtained results showed a lower concentration of aluminum (2.6%) relative to the base FeCrAl fiber elemental composition of 5.76%.

Furthermore, EDS analyses of the 17 μm fiber were conducted on a line covering the diameter, including the oxide layer, as shown in Figure 10c. The EDS results along this line show a high concentration of Al and O with a low concentration of Fe at the oxide layer region on both edges of the fiber. Besides, a drop in the concentration of the Al element is observed inside the fiber.

**Figure 11:**
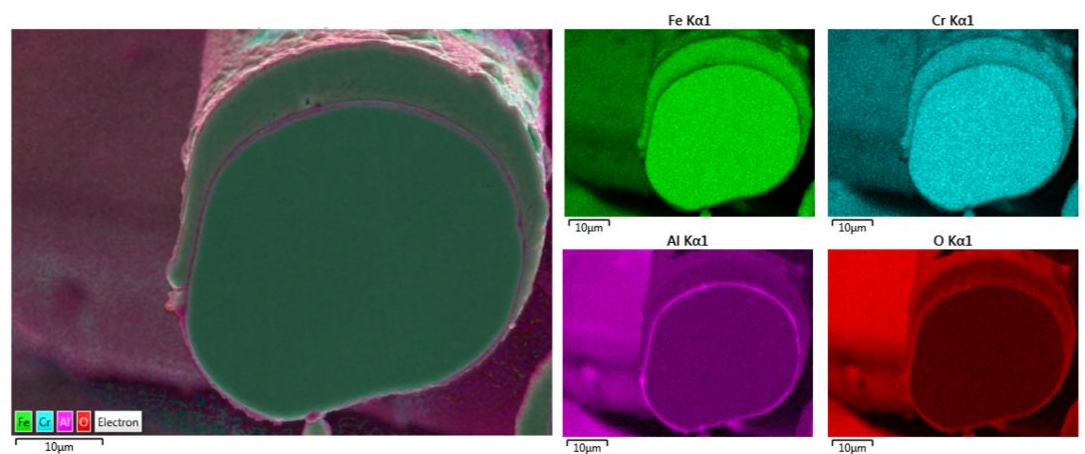
Elemental EDS mapping of Fe, Cr, Al, and O of a 17 μm FeCrAl fiber after one MSHT cycle.

Additional elemental EDS analyses of the 17 μm fiber’s entire cross-section area after one MSHT cycle are shown in Figure 11. Similar to the results obtained for the 12 μm fiber, the elemental mapping after one MSHT cycle of the 17 μm fiber presents a homogenous FeCrAl alloy of the fiber and confirms the formation of an oxide layer around the fiber. The line EDS scans after one MSHT cycle for both the 12 and 17 μm also show comparable results.

#### 3.2.3. After Multiple MSHT Cycles

**Figure 12:**
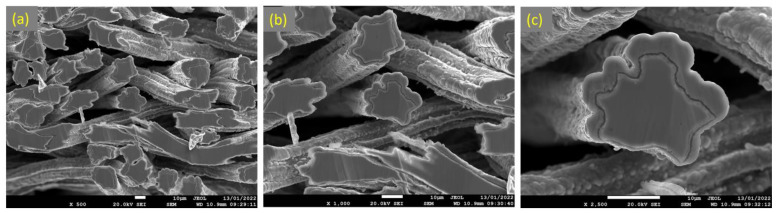
SEM micrographs of a cross-section of 12 μm FeCrAl fibers, after 18 MSHT cycles, at different magnifications: (**a**) ×500, (**b**) ×1000, and (**c**) ×2500.

This section discusses the effect of multiple heat treatment cycles on the development of the oxide layer on the surface of small-diameter FeCrAl fibers. Figure 12a–c shows SEM micrographs to varying magnifications of the cross section of the 12 μm fibers after 18 MSHT cycles. It is evident from Figure 12a–c that the 12 μm fibers developed a thick layer (~4 μm) over the surface of the fibers after 18 MSHT cycles. Besides, the cross-sectional area of the fibers is severely distorted after implementing 18 MSHT cycles, as shown in Figure 12c.

**Figure 13:**
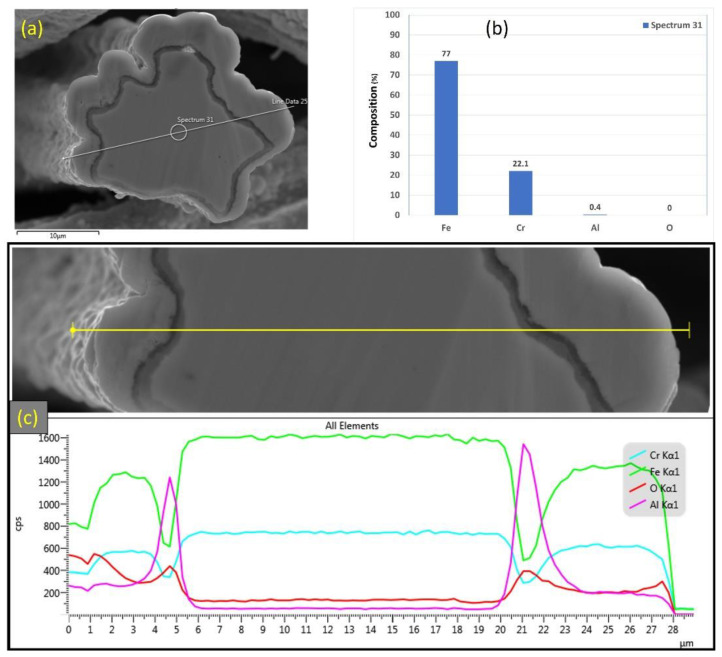
(**a**) SEM micrograph of a cross section of the 12 μm FeCrAl fiber after 18 MSHT cycles, (**b**) EDS scan at the center of the fiber, (**c**) the characteristic X-ray counts along the specified line scan (Line Data 25) marked in (**a**).

EDS analyses were also conducted on a 12 μm fiber, as shown in Figure 13a–c. Figure 13b depicts the EDS spectrum for a small circular area close to the center of the 12 μm fiber. The results revealed a low aluminum concentration (0.4%) relative to the base FeCrAl fiber elemental composition of 5.76%. Moreover, EDS analyses were repeated on a line covering the fiber’s entire diameter with its oxide layer, as shown in Figure 13c. Similar to the results obtained after one MSHT cycle, the EDS line scan results showed a high concentration of Al and O and a low concentration of Fe at the oxide layer region on both edges of the fiber. In addition, a sharp increase in the concentration of Al was observed between the base metal fiber and the oxide-scale layer. These findings show the continuous outward diffusion of aluminum from the base metal alloy to the fiber’s surface to develop the oxide layer after multiple MSHT cycles. The growth of the oxide layer on the 12 μm fiber’ surface explains the high weight gain percentage observed in the TGA analyses after 18 MSHT cycles. 

**Figure 14:**
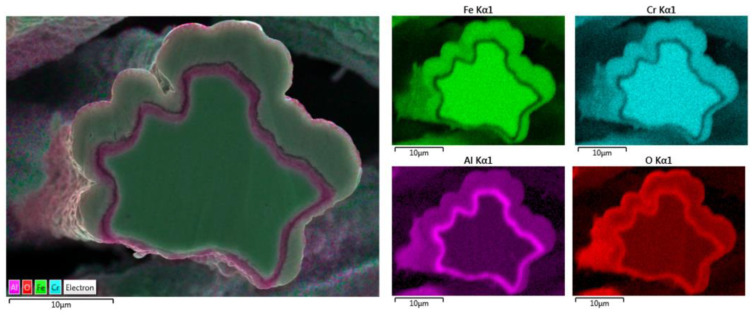
Elemental EDS mapping of Fe, Cr, Al, and O of a 12 μm FeCrAl fiber after 18 MSHT cycles.

Elemental EDS analyses of the entire cross-section area of the 12 μm fiber after 18 MSHT cycles are shown in Figure 14. The results show the depletion of Al from the FeCrAl base metal after the 18 MSHT cycles. In addition, the elemental mapping of Al and O illustrates the development of a thick oxide layer around the fiber’s surface. Additionally, a higher concentration of Al and O is observed between the base metal fiber and the oxide-scale layer, indicating high outward diffusion of aluminum from the base metal alloy.

**Figure 15:**
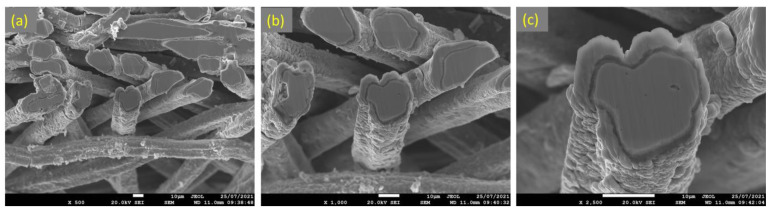
SEM micrographs of a cross section of 17 μm FeCrAl fibers, after 18 MSHT cycles, at different magnifications: (**a**) ×500, (**b**) ×1000, and (**c**) ×2500.

**Figure 16:**
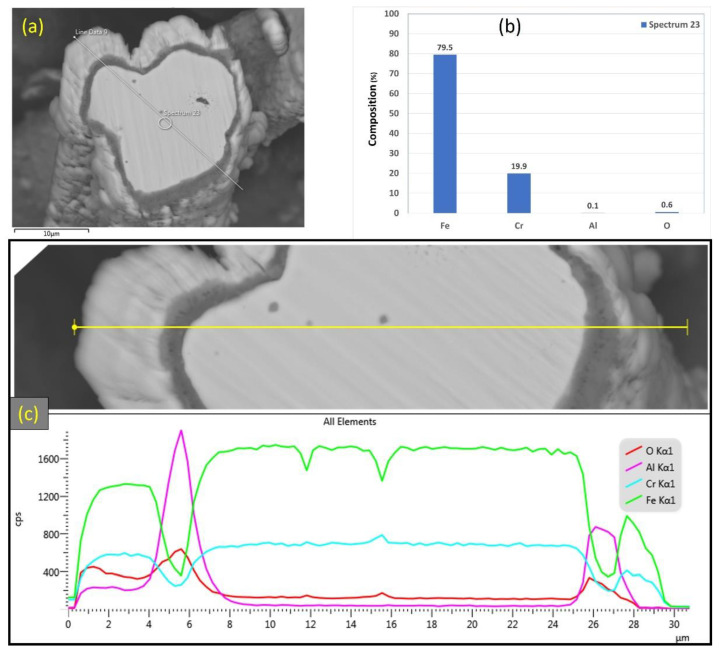
(**a**) SEM micrograph of a cross-section of the 17 μm FeCrAl fiber after 18 MSHT cycles, (**b**) EDS scan at the center of the fiber, (**c**) the characteristic X-ray counts along the specified line scan (Line Data 9) marked in (**a**).

Similarly, Figure 15a–c depicts SEM images at various magnifications of the cross section of the 17 μm fibers after performing 18 MSHT cycles. As shown in Figure 15, the 17 μm fibers formed a thick layer (~5 μm) over the surface of the fibers after 18 MSHT cycles. Moreover, the original circular cross-sectional area of the fibers is significantly distorted after executing 18 MSHT cycles, as shown in Figure 15c. Figure 16 shows EDS analyses conducted on the cross section of a 17 μm fiber. Figure 16b depicts the EDS spectrum for a point near the center of the 17 μm fiber. The results showed a significantly low aluminum concentration near the center of the fiber compared with the base FeCrAl fiber elemental composition.

After 18 MSHT cycles, the EDS analyses were also conducted on a line covering the 17 μm fiber’s entire diameter, including the oxide layer (Figure 16c). Comparable results to Figure 13c for the 12 μm fiber were obtained for the 17 μm fiber, showing high concentrations of Al and O and a low concentration of Fe at the oxide layer region on both edges of the fiber. Furthermore, a sharp increase in the concentration of Al was detected between the base metal fiber and the oxide-scale layer. These results confirm the outward diffusion of aluminum from the fiber base metal alloy to the surface to develop the oxide layer even after multiple MSHT cycles.

**Figure 17:**
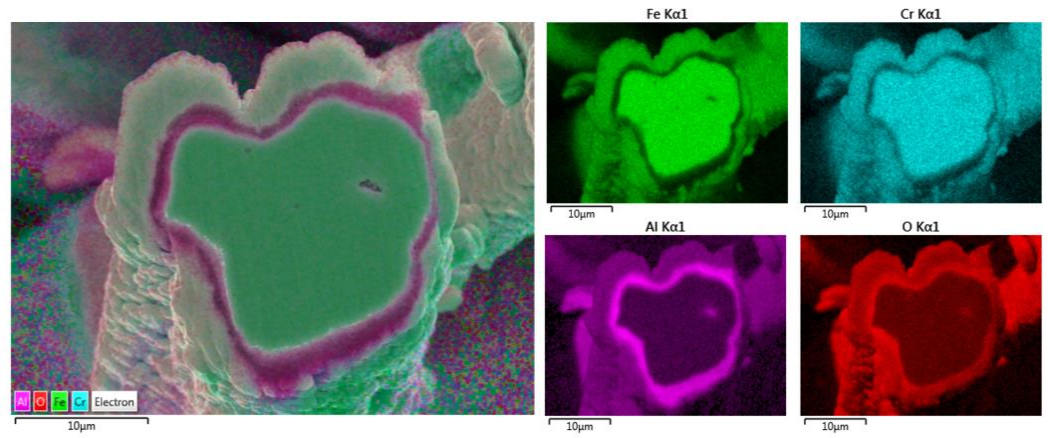
Elemental EDS mapping of Fe, Cr, Al, and O of a 17 μm FeCrAl fiber after 18 MSHT cycles.

Additionally, the elemental EDS mapping was repeated on the 17 μm fiber’s cross-section area after 18 MSHT cycles, as shown in Figure 17. The elemental mapping demonstrates a lack of Al and O within the inner surface of the FeCrAl fiber after the multiple heat treatment cycles. Furthermore, the elemental mapping of Al and O reveals the formation of the Al_2_O_3_ oxide layer around the fiber’s surface. In addition, a higher concentration of Al and O is detected between the base metal fiber and the oxide-scale layer.

**Figure 18:**
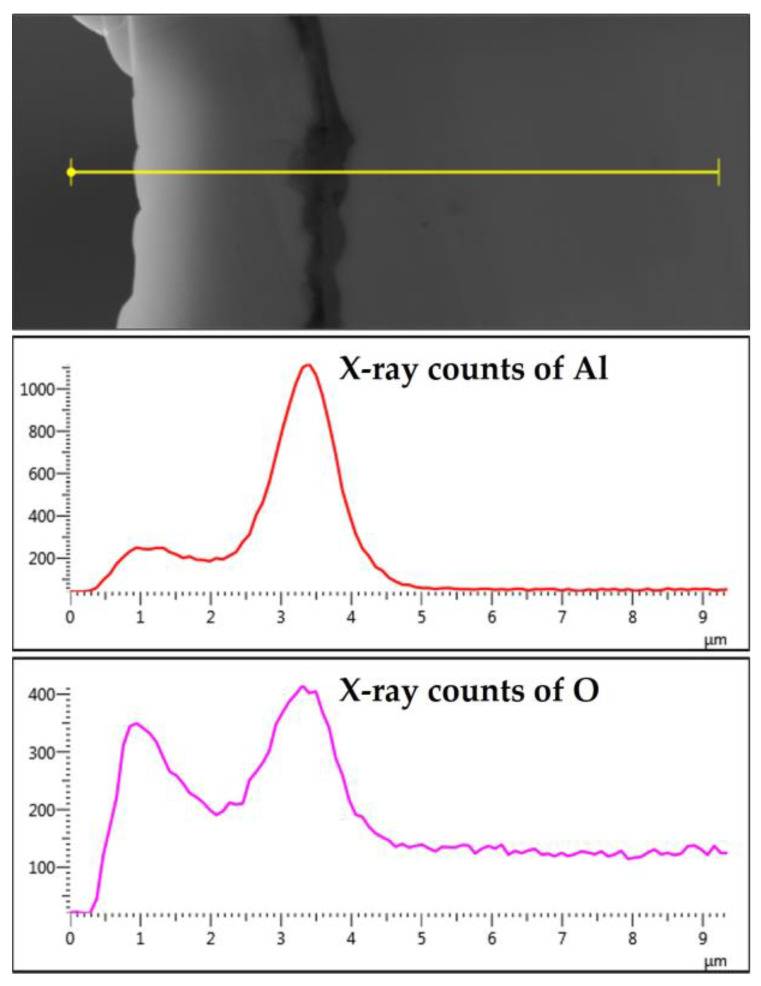
The characteristic X-ray counts of Al and O along a line starting from the outer edge of the oxide-scale layer into a few microns of the 12 μm fiber after 18 MSHT cycles.

The elemental EDS mapping of the 12 and 17 μm fibers shows aluminum accumulation in a thin layer between the base metal alloy and the oxide-scale layer. The aluminum accumulation is thin but noticeable after the first MSHT, forming an interface layer trapped under the oxide-scale layer. The high aluminum concentration interface layer becomes thicker and more noticeable after multiple MSHT cycles. Figure 18 depicts the X-ray EDS line scan count data of Al and O along a line that covers the oxide-scale layer and a few micrometers of the 12 μm base metal fiber after 18 MSHT cycles. This EDS line scan reveals the high-concentration aluminum at the interface layer, which confirms the high outward diffusion of aluminum from the base metal alloy. The existence of this interface layer with high aluminum concentration can be explained by the fact that the rate of outward aluminum diffusion is higher than the rate of oxide-scale layer growth. On the other hand, the 40 μm fibers’ elemental EDS mapping and line scans after one MSHT or multiple MSHT cycles, reported by Ibrahim et al. [36], did not show the existence of a high-concentration interface layer.

Compared with the 12 and 17 μm fibers, Ibrahim et al. [36] showed that the 40 μm fibers require multiple MSHT cycles to form a thick and uniform oxide layer on their surface. This confirms the slow outward diffusion of aluminum during the heat treatment from the 40 μm fibers compared with the 12 and 17 μm fibers.

**Figure 19:**
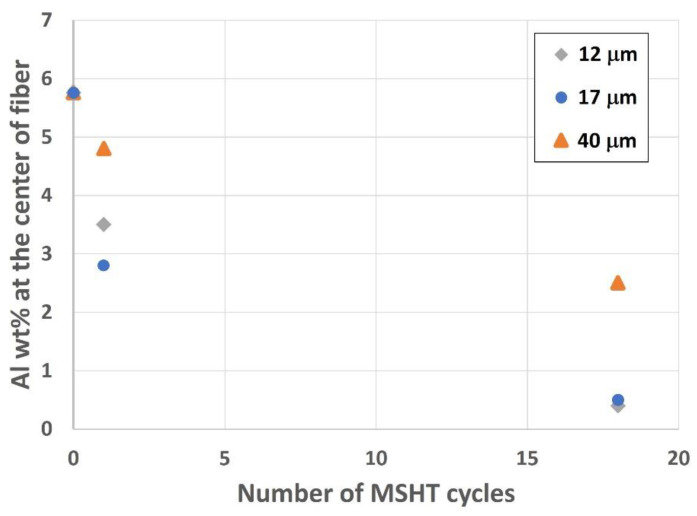
Aluminum concentration, in wt.%, at the center of the 12, 17, and 40 μm FeCrAl fibers versus the number of MSHT cycles.

Analyzing the center of the fibers is vital to exploring the depletion of aluminum from the different FeCrAl fibers. Figure 19 shows the effect of MSHT cycles on aluminum concentration, average in weight percent (wt.%) from various measurements, at the center of small-diameter (12 and 17 μm) and large-diameter (40 μm) FeCrAl fibers. The drop in aluminum concentration at the center of the fiber is significantly higher for the small-diameter fibers (12 and 17 μm) compared with the 40 μm fiber after multiple MSHT cycles. This is because the sizes of aluminum reservoirs of the 12 and 17 μm fibers are much smaller, and the surface areas are much larger than the 40 μm fibers. Therefore, the smaller-diameter FeCrAl fibers allow for more outward diffusion of aluminum to the fiber’s surface, resulting in faster aluminum depletion from the base metal reservoir than the large-diameter FeCrAl fiber. 

In summary, the SEM and EDS results revealed that the size affects the formation and development of the oxide layer around the fiber. Compared with the 40 μm fiber, the small-diameter fibers, 12 and 17 μm, developed a distinctive interface layer with high aluminum concentration and experienced severe distortion of the original circular cross-section area after performing single and multiple MSHT cycles. 

### 3.3. X-ray Diffraction (XRD) Analyses

**Figure 20:**
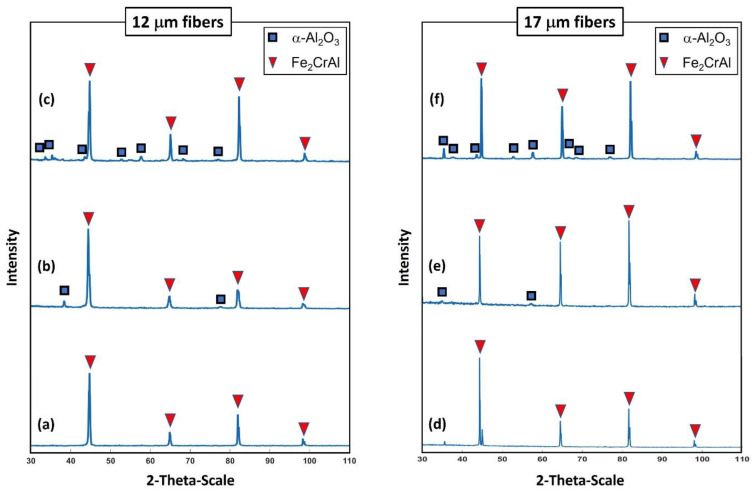
XRD test shows the crystalline pattern: For the 12 μm fibers, (**a**) without heat treatment, (**b**) after one MSHT cycle, and (**c**) after 18 MSHT cycles. For the 17 μm fibers, (**d**) without heat treatment, (**e**) after one MSHT cycle, and (**f**) after 18 MSHT cycles.

Examinations of the crystallographic structure on the surface for the 12 and 17 μm fibers after different heat treatment conditions were accomplished using XRD analyses. Figure 20a–f depicts two-dimensional XRD patterns of the 12 and 17 μm fibers before heat treatment, after one MSHT cycle, and after 18 MSHT cycles. The 12 and 17 μm fibers exhibited similar diffraction patterns at all heat treatment conditions. Similar results were also reported by Ibrahim et al. [36] for the 40 μm fibers. For the case of no heat treatment, the peaks of the XRD patterns for the two fibers corresponding to the crystalline Fe_2_CrAl phase are shown in Figure 20a,d. It should be noted that no stable diffraction pattern peaks of α-Al_2_O_3_ were detected for both fibers before the heat treatment cycle—illustrating that the outward diffusion of the Al forms an amorphous layer on the surface of the FeCrAl fibers. However, for the case after one MSHT cycle, diffraction pattern peaks that correspond to the crystalline Fe_2_CrAl phase and small peaks that correspond to α-Al_2_O_3_ were detected for the 12 and 17 μm fibers (see Figure 20b,e).

For the case after 18 MSHT cycles, the two fibers exhibited XRD pattern peaks of the Fe_2_CrAl phase and α-Al_2_O_3_ crystalline structure, as shown in Figure 20c,f. The XRD results indicate that corundum nucleation and stable α-Al_2_O_3_ layer formation happen after multiple MSHT cycles. Furthermore, after 18 MSHT cycles, the results display the diffraction pattern peaks of the Fe_2_CrAl phase for both fibers. This illustrates that the formation of a thick α-Al_2_O_3_ oxide layer on the surface of the FeCrAl fibers did not affect the crystallographic structure of the metal fibers even after multiple MSHT cycles.

## 4. Concluding Remarks

In the current investigation, two sizes of small-diameter FeCrAl fibers, 12 and 17 μm, were examined at different heat treatment conditions to explore the effect of fiber size on forming a protective oxide layer. In addition, TGA analyses were conducted to measure the percentage of weight change during the development of the oxide layer on the surface of the fibers. The percentage of weight change by the different FeCrAl fibers during the heat treatment process is attributed to the formation and growth of an oxide layer on the surface of the fibers. The TGA results revealed that the percentage of weight gain increases with the decrease in the fiber’s diameter due to the increase in the surface-area-to-weight ratio. The high surface area allows for a more outward diffusion of aluminum to the surface of the fibers to form the alumina oxide layer during the heat treatment cycles.

It was observed that the small-diameter fibers, 12 and 17 μm, developed a thick oxide layer after multiple heat treatment cycles. It was also observed that the FeCrAl fibers with small diameters suffered from severe distortion of the original near-circular cross-section area after multiple MSHT cycles. Furthermore, the elemental EDS mapping showed that the small-diameter fibers formed a thin layer of high aluminum concentration between the base metal fiber and the oxide-scale layer after the first MSHT cycle. The accumulation of aluminum in the interface layer increases after multiple MSHT cycles, developing into a thick layer of high aluminum concentration. The high growth rate of the oxide-scale layer and the limited aluminum reservoir of the small-diameter fibers lead to faster depletion of aluminum from the base metal alloy and irregular growth of the protective oxide layer—limiting the lifetime and durability of the smaller-diameter fibers in high-temperature applications.

## Figures and Tables

**Table 1:** The elemental chemical compositions of the FeCrAl sintered fibers [36].

Element	Chemical Composition (%)
Cr	20.580
Al	5.760
Mn	0.160
Cu	0.046
Ti	0.041
C	0.033
P	0.015
S	0.002
N	0.010
Si	0.240
Fe	Balance

**Table 2:** Multistage thermal oxidation (MSHT) cycle.

Heat Treatment Stage	Temperature (°C)	Time (h)	Heating Rate (°C/min)
1	930	1	50
2	960	1	50
3	990	2	50

**Table 3:** Percentage of weight gains during each stage of the first MSHT cycle for the 12 and 17 µm FeCrAl fibers.

Fiber Size (µm)	Weight Gained (%)		
	First Stage (930 °C for 1 h)	Second Stage (960 °C for 1 h)	Third Stage (990 °C for 2 h)
12	1.0	0.6	0.8
17	0.7	0.3	0.5

**Table 4:** Estimation of the surface area per weight for different sizes of the FeCrAl fibers.

Diameter (μm)	Surface Area per Weight (cm^2^/g)
12	466
17	329
40	140
